# MEETINGS CALENDAR - 2017

**Published:** 2017

## June

### ► 1 - Toronto Update on Lung Transplantation and Artificial Lung Support

Toronto, Canada

Informations: Nancy Bush

Phone: 4169782719

E-mail: info-SUR1726@cpdtoronto.ca

Site: www.cpd.utoronto.ca/lungtransplant/

### ► 2 and 3 - Thoracic Refresher

Toronto, Canada

Informations: Nancy Bush

Phone: 4169782719

E-mail: info-SUR1726@cpdtoronto.ca

Site: www.torontothoracicrefresher.ca/

### ► 5 to 9 - Fundamentals in Cardiac Surgery: Part II

Windsor, United Kingdom

Informations: EACTS

E-mail: info@eacts.co.uk

Site: www.eacts.org/academy/2017-programme/

### ► 12 to 14 - Thoracic Surgery: Part II

Windsor, United Kingdom

Informations: EACTS

E-mail: info@eacts.co.uk

Site: www.eacts.org/academy/2017-programme/

### ► 14 to 17 17^th^ European Congress on Extracorporeal Circulation Technology

Marseille, France

Informations: Judith Moonen

Phone: + 31 6 29 229 655

E-mail: office@fecect.org

Site: www.fecect.org

### ► 15 to 17 - Ventricular Assist Device Co-ordinators Training Course

Berlin, Germany

Informations: EACTS

E-mail: info@eacts.co.uk

Site: www.eacts.org/academy/2017-programme/

### ► 19 to 21 - 18^th^ Annual Cardiologists Conference

Paris, France

Informations: Raul Wilson

Site: annualmeeting.conferenceseries.com/cardiologists/

### ► 20 and 21 - Magna Græcia AORtic Interventional Project^®^ (MAORI) 5^th^ Symposium Complex Diseases of Thoracic and Thoraco-Abdominal Aorta

Catanzaro, Italy

Informations: Prof.Pasquale

Phone: +39 09613695851

E-mail: umgaorta@unicz.it

Site: maori.unicz.it/

### ► 21 to 24 - WTSA 43^rd^ Annual Meeting

Colorado Springs, United States

Informations: Heather Nutter

E-mail: meetings@westernthoracic.org

Site: meetings.westernthoracic.org/

### ► 21 to 24 - ASAIO 63^rd^ Annual Conference

Chicago, United States

Informations: ASAIO Headquarters

Site: www.asaio.com

### ► 28 to 1 - The New Orleans Conference - Las Vegas Edition

Las Vegas, United States

Informations: Joseph Basha

Phone: 318-623-0890

Site: www.TheNewOrleansConference.com

## July

### ► 30 and 1 - Basic Science

Windsor, United Arab Emirates

Informations: EACTS

E-mail: info@eacts.co.uk

Site: www.eacts.org/academy/2017-programme/

### ► 24 and 25 - International Conference on Vascular Biology & Medicine

Chicago, United States

Informations: Suzanne Williams

Site: vascular.cmesociety.com/

## August

### ► 14 to 16 - 4^th^ International Conference on Brain Disorder and Brain Injury

Toronto, Canada

Informations: Karlin Zoe

Phone: 7025085200

Fax: 7025085200

E-mail: braininjury2017@protonmail.com

Site: http://braininjury.alliedacademies.com/

### ► 18 to 20 - International Coronary Congress

New York, United States

Informations: EJ Weldon

Phone: 978-927-8330

E-mail: info@InternationalCoronaryCongress.com

Site: http://InternationalCoronaryCongress.com

### ► 24 to 27 - 4^th^ HeartCares

Informations:

Phone: 662-547-0930

Fax: 662-589-9321

E-mail: cdihearts@gmail.com

Site: http://www.heartcareheart.org

## September

### ► 1 to 3 - 27^th^ Annual Congress of the World Society of Cardiovascular &Thoracic Surgeons

Astana, Kazakhstan

Informations: Stella Papandreopoulou

E-mail: infowscts@gmail.com

Site: www.wscts.net

### ► 8 and 9 - 11^th^ Current Trends in Aortic, Cardiac and General Thoracic Surgery

Houston, United States

Informations: Alan P. Stolz, M.Ed.

Phone: 832-355-9930

Fax: 832-355-9931

E-mail: astolz@bcm.edu

Site: www.cme.texasheart.org

### ► 11 to 13 - 2^nd^ International Conference on Hypertension & Healthcare

Amsterdam, Netherlands

Informations: Jessie Alison

Phone: 7025089022

Fax: 7025089022

### ► 13 to 16 - ATSOP: Advances in Thoracic Surgery, Oncology and Pulmonology

Singapore, Singapore

Informations: Eva

Site: https://www.ATSOP.sg

### ► 14 to 17 - Birmingham Review Course in Cardiothoracic Surgery

Birmingham, United Kingdom

Informations: L.R. Associates - Ms. L. Richardson

Phone: 01296 733 823

Fax: 01296 733 823

E-mail: lorrainerichardson1@btinternet.com

### ► 15 - Current Paradigms in Interventional Pulmonology: Endobronchial Ultrasound and Lung Cancer Staging

New York, United States

Informations: Office of Continuing Medical Education

Phone: 646-227-2025

E-mail: cme@mskcc.org

Site: www.mskcc.org/IPcourse

### ► 18 and 19 - Annual Conference on Heart Diseases Toronto, Canada

Informations: Kathie Johnson

Phone: 8282143944

E-mail: heartdiseases@alliedconferences.org

Site: http://heartdiseases.alliedacademies.com/

### ► 21 to 24 - 13^th^ Annual Meeting of the Euro-Asian Bridge Society

Iaşi, Romania

Informations:

Site: https://www.eab2017romania.org/

### ► 21 and 22 - STS/EACTS Latin America Cardiovascular

Surgery Conference Cartagena, Colombia

Informations: Damon Marquis

Phone: 312-202-5800

Fax: 312-202-5801

E-mail: info@CardiovascularSurgeryConference.org

Site: http://www.cardiovascularsurgeryconference.org

### ► 21 to 23 - 2017 Duke Masters of Minimally Invasive Thoracic Surgery

Orlando, United States

Informations: Lindsay Oppedisano

Phone: 800-253-7657; 203-263-0006 ext 120

Fax: 203-263-4839

E-mail: loppedisano@cine-med.net

Site: innovation.surgery.duke.ed/courses

### ► 21 and 22 - 2017 International Conference and Expo on Heart Surgery

San Antonio, United States

Informations: Daniella Mosby

Phone: 4084292646

E-mail: heartsurgery@cmesocietyconferences.com

Site: http://heartsurgery.cmesociety.com/

### ► 21 to 23 - Cases & Controversies in Cardiovascular Disease

Williamsburg, United States

Informations: Carol Ann Rosenberg

Phone: 434-924-5310

E-mail: uvacme@virginia.edu

Site: www.cmevillage.com

### ► 25 to 27 - ESTS Course on Chest Wall Surgery

Hamburg, Germany

Informations: Sue Hesford

E-mail: sue@ests.org.uk

Site: www.ests.org

### ► 28 to 30 -13^th^ International Conference on Pediatric Mechanical Circulatory Support Systems and Pediatric Cardiopulmonary Perfusion

Rome, Italy

Informations: Akif Ündar, PhD

Phone: 717-379-5020

Fax: 717-531-0355

E-mail: congressi@opbg.net

Site: https://www.ispmcs.org/annual-conference/

### ► 28 to 30 - XIX National Congress of "Societa' Italiana di Endoscopia Toracica"

Lecce, Italy

Informations: Gaetano Di Rienzo, MD, FETCS

Phone: +39/349/6398344

Fax: +39/832/661608

E-mail: gaetanodirienzo1@gmail.com

Site: http://www.endoscopiatoracica.it

